# *FGFR2* gene amplification and clinicopathological features in gastric cancer

**DOI:** 10.1038/bjc.2011.603

**Published:** 2012-01-12

**Authors:** K Matsumoto, T Arao, T Hamaguchi, Y Shimada, K Kato, I Oda, H Taniguchi, F Koizumi, K Yanagihara, H Sasaki, K Nishio, Y Yamada

**Affiliations:** 1 Department of Genome Biology, Kinki University Faculty of Medicine, Osaka 589-8511, Japan; 2 Gastrointestinal Medical Oncology, National Cancer Center Hospital, Tokyo 104-0045, Japan; 3 Endoscopic Division, National Cancer Center Hospital, Tokyo 104-0045, Japan; 4 Pathology Division, National Cancer Center Hospital, Tokyo 104-0045, Japan; 5 Shien Lab, National Cancer Center Hospital, Tokyo 104-0045, Japan; 6 Department of Life Sciences, Yasuda Women's University Faculty of Pharmacy, 6-13-1, Ando, Asaminami, Hiroshima 731-0153, Japan; 7 Division of Genetics, National Cancer Center Research Institute, Tokyo 104-0045, Japan

**Keywords:** *FGFR2*, gastric cancer, gene amplification

## Abstract

**Background:**

Frequency of *FGFR2* amplification, its clinicopathological features, and the results of high-throughput screening assays in a large cohort of gastric clinical samples remain largely unclear.

**Methods:**

Drug sensitivity to a fibroblast growth factor receptor (FGFR) inhibitor was evaluated *in vitro*. The gene amplification of the *FGFRs* in formalin-fixed, paraffin-embedded (FFPE) gastric cancer tissues was determined by a real-time PCR-based copy number assay and fluorescence *in situ* hybridisation (FISH).

**Results:**

*FGFR2* amplification confers hypersensitivity to FGFR inhibitor in gastric cancer cell lines. The copy number assay revealed that 4.1% (11 out of 267) of the gastric cancers harboured *FGFR2* amplification. No amplification of the three other family members (*FGFR1*, *3* and *4*) was detected. A FISH analysis was performed on 7 cases among 11 *FGFR2*-amplified cases and showed that 6 of these 7 cases were highly amplified, while the remaining 1 had a relatively low grade of amplification. Although the difference was not significant, patients with *FGFR2* amplification tended to exhibit a shorter overall survival period.

**Conclusion:**

*FGFR2* amplification was observed in 4.1% of gastric cancers and our established PCR-based copy number assay could be a powerful tool for detecting *FGFR2* amplification using FFPE samples. Our results strongly encourage the development of FGFR-targeted therapy for gastric cancers with *FGFR2* amplification.

Intensive investigations of anticancer treatments for gastric cancer have been done over the past three decades; however, the prognosis for patients with unresectable advanced or recurrent gastric cancer remains poor (Bittoni *et al*, 2010; Fujii *et al*, 2010), and new therapeutic modalities are needed.

Fibroblast growth factors (FGFs) and their receptors are considered to be associated with multiple biological activities, including fundamental developmental pathways, cellular proliferation, differentiation, motility and transforming activities (Itoh *et al*, 1994; Moffa *et al*, 2004; Grose and Dickson, 2005). Fibroblast growth factor signalling is also involved in many physiological roles in the adult organism, such as the regulation of angiogenesis and wound repair, and FGF receptors (FGFRs) are expressed on many different cell types and regulate key cell behaviours of cancer cells (Turner and Grose, 2010). Emerging evidence has demonstrated that the deregulation of FGF signalling is frequently observed in various solid cancers and haematological malignancies (Beenken and Mohammadi, 2009). The most well-known association with *FGFR* mutations is the *FGFR3* mutation observed in bladder cancer, in which somatic mutations in coding regions are observed in about 50% of all specimens (Cappellen *et al*, 1999; Turner and Grose, 2010). Other genetic alterations in *FGFR3* include gene amplification in bladder cancer and translocation in myeloma (Turner and Grose, 2010). Similarly, the deregulation of FGF signalling has been reported in various malignancies. Glioblastoma exhibits FGFR1 kinase domain gain-of-function mutations, and FGFR1 is abnormally activated in malignant prostate cells. In 8p11 myeloproliferative syndrome, translocations fuse different proteins in frame with the FGFR1 kinase domain, causing the constitutive dimerisation of the kinase (Giri *et al*, 1999; Rand *et al*, 2005; Beenken and Mohammadi, 2009). The *FGFR1* amplification has been reported in approximately 10% of breast cancers (Courjal *et al*, 1997) and oral squamous carcinomas, and has been also found at a low incidence in ovarian cancer, bladder cancer and rhabdomyosarcoma (Turner and Grose, 2010). *FGFR2* mutations are observed in 12% of endometrial cancers but are reportedly rare in gastric cancers (Jang *et al*, 2001; Dutt *et al*, 2008). The *K-sam* gene was first identified and characterised as an amplified gene in the human gastric cancer cell line KATO-III (Hattori *et al*, 1990; Ueda *et al*, 1999), and its product was later found to be identical to the bacteria-expressed kinase, or keratinocyte growth factor receptor, and FGF receptor 2 (FGFR2). *FGFR2* amplification has been found in diffuse-type gastric cancer-derived cell lines and the amplification was preferentially detected in diffuse-type gastric cancer. FGFR2 protein overexpression was detected using immunohistochemical staining in 20 of 38 advanced cases of diffuse-type gastric cancer (Hattori *et al*, 1996). FGFR2 protein expression was observed in 31% of the gastric carcinomas and was positively correlated with scirrhous cancer, a diffuse type, the invasion depth, the infiltration type and a poor prognosis (Toyokawa *et al*, 2009).

On the other hand, along with another group, we previously reported that *FGFR2* amplification confers hypersensitivity to FGFR inhibitor in gastric cancer cell lines both *in vitro* and *in vivo* (Nakamura *et al*, 2006; Takeda *et al*, 2007), strongly suggesting that *FGFR2* amplification may be a promising molecular target for the treatment of *FGFR2*-amplified gastric cancer. However, very limited information on *FGFR2* amplification is available regarding the frequency, the degree of the increase in the copy number, the histology and a high-throughput screening method in gastric cancer. In this report, we retrospectively studied these issues using formalin-fixed, paraffin-embedded (FFPE) samples in patients with gastric cancer who underwent surgery in an attempt to advance FGFR2-targeted therapy for gastric cancer.

## Materials and methods

### Cell culture

All of the gastric cancer cell lines used in this study were maintained in RPMI-1640 medium (Sigma, St Louis, MO, USA), except for IM95 (DMEM; Nissui Pharmaceutical, Tokyo, Japan), supplemented with 10% heat-inactivated fetal bovine serum (Gibco BRL, Grand Island, NY, USA), penicillin and streptomycin in a humidified atmosphere of 5% CO_2_ at 37 °C. IM95 and OCUM1 were obtained from the Japanese Collection of Research Bioresources (Osaka, Japan) and the others were provided from National Cancer Center Research Institute (Tokyo, Japan).

### Patients

A total of 267 patients with histologically confirmed gastric cancer who had undergone surgery at the National Cancer Center Hospital between 1996 and 2006 were included in this study. All the patients in this series had an Eastern Cooperative Oncology Group performance status of 0 to 2 and had undergone surgery. Of these patients, one subject was excluded because an insufficient quantity of DNA was extracted from the patient's specimen. Thus, samples from the remaining 267 patients were analysed. This study was approved by the institutional review board of the National Cancer Center Hospital.

### Isolation of genomic DNA

Genomic DNA samples were extracted from surgical specimens preserved as FFPE tissue using a QIAamp DNA Micro kit (Qiagen, Hilden, Germany) according to the manufacturer's instructions. Macro-dissection of the FFPE samples was performed to select a cancer region, which was marked by a pathologist after deparaffinisation. The DNA concentration was determined using the NanoDrop2000 (Thermo Scientific, Waltham, MA, USA).

### Real-time reverse-transcription PCR (RT–PCR)

cDNA was prepared from the total RNA of each cultured cell line using a GeneAmp RNA-PCR kit (Applied Biosystems, Foster City, CA, USA). Real-time RT–PCR amplification was carried out using a Thermal Cycler Dice (Takara, Otsu, Japan) in accordance with the manufacturer's instructions under the following conditions: 95 °C for 5 min, and 50 cycles of 95 °C for 10 s and 60 °C for 30 s. The primers used for the real-time RT–PCR were as follows: *FGFR2*, forward 5′-GATAAATACTTCCAATGCAGAAGTGCT-3′ and reverse 5′-TGCCCTATATAATTGGAGACCTTACA-3′ *GAPDH*, forward 5′-GCACCGTCAAGGCTGAGAAC-3′ and reverse 5′-ATGGTGGTGAAGACGCCAGT-3′. *GAPDH* was used to normalise the expression levels in the subsequent quantitative analyses.

### Immunoblotting

A western blot analysis was performed as described previously (Matsumoto *et al*, 2009). The following antibodies were used: monoclonal FGFR2 antibody (Santa Cruz Biotechnology, Santa Cruz, CA, USA), *β*-actin antibody and HRP-conjugated secondary antibody (Cell Signaling Technology, Beverly, MA, USA).

### Cell growth inhibitory assay

To evaluate growth inhibition in the presence of various concentrations of PD173074 (Sigma), we used an MTT assay and a previously described method (Kaneda *et al*, 2010). Briefly, the cells were seeded at a density of 2 × 10^3^ cells per well in 96-well plates. After 24 h, PD173074 was added and the incubation was further continued for 72 h at 37 °C. The assay was conducted in triplicate.

### Copy number assay for four FGFR family genes

The copy numbers for *FGFR 1–4* were determined using commercially available and pre-designed TaqMan Copy Number Assays according to the manufacturer's instructions (Applied Biosystems). The primer IDs used for *FGFRs* were as follows: *FGFR1,* Hs02862256_cn; *FGFR2,* HS05182482_cn (intron 14) and Hs05114211_cn (intron 12); *FGFR3,* Hs03518314_cn; and *FGFR4*, Hs01949336_cn. The *TERT* locus was used for the internal reference copy number. Human Genomic DNA (Takara) was used as a normal control. Real-time genomic PCR was performed in a total volume of 20 *μ*l in each well, containing 10 *μ*l of TaqMan genotyping master mix, 20 ng of genomic DNA and each primer. The PCR conditions were 95 °C for 10 min and 40 cycles of 95 °C for 15 s and 60 °C for 1 min; the resulting products were detected using the ABI PRISM 7900HT Sequence Detection System (Applied Biosystems). Data were analysed using SDS 2.2 software and CopyCaller software (Applied Biosystems).

### Fluorescence *in situ* hybridisation analysis

The fluorescence *in situ* hybridisation (FISH) method was previously descried (Motoi *et al*, 2010). Probes designed to detect the *FGFR2* gene and the *CEN10p* on chromosome 10 were labelled with fluorescein isothiocyanate or Texas red and were designed to hybridise to the adjacent genomic sequence spanning approximately 0.33 and 0.64 Mb, respectively. The probes were generated from appropriate clones from a library of human genomic clones (GSP Laboratory, Kawasaki, Japan). Deparaffinised tissue sections were air dried and pre-treated with the GSP paraffin pre-treatment kit (GSP Laboratory). In all, 10 *μ*l of fluorescent FISH probe was heated for 5 min at 73–75 °C in a waterbath for denaturation. The tissue sections were then placed in a denaturant solution (70% formamide/2 × saline sodium citrate (SSC) pH 7-8) in a 73–75 °C waterbath, denatured for 5 min, dehydrated in 70 and 100% ethanol for 1 min each at room temperature, and air-dried. Denatured probes were applied, and the specimens were covered with a coverglass and placed on a heated block at 45–50 °C. Then, the slides were sealed with rubber cement and placed in a pre-warmed humidified box overnight at 37 °C. Stringent washing was performed using 2 × SSC/0.3% NP-40 at room temperature and at 72 °C for 5 min and then with 2 × SSC at room temperature. The signals were observed using fluorescence microscopy, and the FISH signals were evaluated by independent observers (TM and AK). After screening all the complete sections, images of the tumour cells were captured and recorded and the signals for 20 random nuclei were counted for an area where individual cells were recognised on at least 10 representative images. The positive result of copy number gain is determined as follows (FGFR2/CEN10p⩾2.0).

### Statistical analysis

The statistical analyses of the clinicopathological features were performed using the Student *t*-test and the *χ*^2^ test using PAWS Statistics 18 (SPSS Japan Inc., Tokyo, Japan). The overall survival (OS) curves were estimated using the Kaplan–Meier method.

## Results

### *FGFR2* amplification confers hypersensitivity to FGFR inhibitor in gastric cancer cell lines

**Figure 1:**
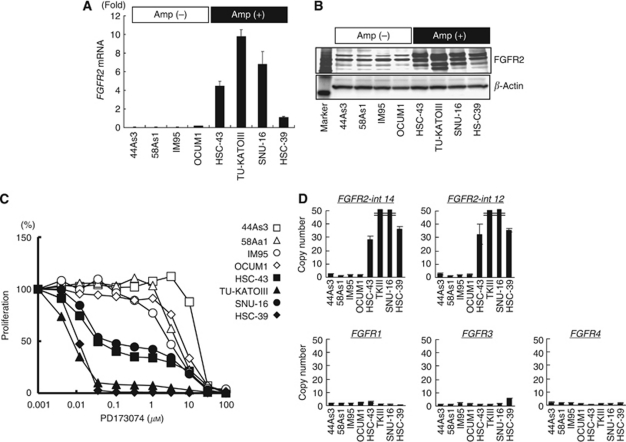
*FGFR2* amplification in gastric cancer cell lines. (**A**) The mRNA expression levels of *FGFR2* were determined using real-time RT–PCR for eight gastric cancer cell lines. *FGFR2* mRNA: normalised mRNA expression levels (*FGFR2*/*GAPDH* × 10^3^). (**B**) Western blot analysis for FGFR2 expression. *β*-Actin was used as an internal control. Marker, molecular marker. (**C**) Growth inhibition assay for the FGFR inhibitor PD173074, evaluated at the indicated concentrations using an MTT assay. (**D**) Evaluation of DNA copy number assay using gastric cancer cell lines. A TaqMan copy number assay was performed to determine the copy number using specific primers for the genomic loci of the *FGFR1–4* genes against DNA samples. Amp, gene amplification. FGFR2-int-14 and FGFR2-int-12, different primers for intron 14 or intron 12 of *FGFR2*.

We examined the growth inhibitory effect of PD173074 (0.004–80 *μ*M) on four *FGFR2*-amplified (HSC-43, TU-KATPIII, SNU-16 and HSC-39) and four non-amplified (44As3, 58As1, IM95 and OCUM1) gastric cancer cell lines. The *FGFR2* amplification status of each cell line had already been examined using a CGH analysis (unpublished data). The mRNA and protein expressions of FGFR2 were overexpressed in the *FGFR2*-amplified cell lines (Figures 1A and B). A growth inhibitory assay showed that the IC_50_ values of the FGFR inhibitor PD173074 in *FGFR2*-amplified cells were 0.01–0.07 *μ*M, whereas those in non-amplified cells were 2.6–13.2 *μ*M, indicating that *FGFR2* amplification conferred an approximately 100-fold hypersensitivity to FGFR inhibitor in gastric cancer cell lines (Figure 1C).

### *FGFR2* amplification in clinical gastric cancer cell lines and surgical specimens

**Figure 2:**
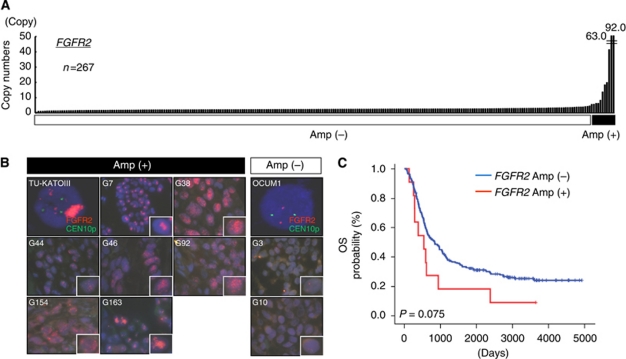
(**A**) Amplification of *FGFRs* in surgical specimens of gastric cancer. A TaqMan copy number assay for *FGFR2* was performed using DNA samples obtained from 267 FFPE samples. Human normal genomic DNA was used as a normal control. *FGFR2* amplification over 5 copies was observed in 11 cases (92.0, 63.0, 41.4, 19.9, 18.4, 13.7, 8.3, 6.2, 6.2, 5.7 and 5.6 copies). (**B**) Fluorescence *in situ* hybridisation analysis of *FGFR2*-amplified KATO-III cells, non-amplified OCUM1 cells and nine surgical specimens of gastric cancer. Green, signal of *CEN10P* locus; Red, signal of *FGFR2* locus; G3∼G92, sample numbers; Amp, gene amplification. High-power images are presented for a single cancer cell. (**C**) Overall survival in *FGFR2*-amplified gastric cancer. Kaplan–Meier curves for OS according to the *FGFR2* amplification status.

To develop a high-throughput method for detecting *FGFR2* gene amplification in a clinical setting, we verified a real-time PCR-based detection method, the TaqMan Copy Number Assay. The *FGFR2* copy number was 1.4–2.7 copies in the four non-amplified cell lines; however, the numbers in the four *FGFR2*-amplified cell lines were 28.2, 231.7, 88.2 and 36.3 copies, respectively (Figure 1D). In addition, another primer in intron 12 of *FGFR2* produced a very similar result (*R*=0.99, Figure 1D). Collectively, these results suggested that a DNA copy number assay for *FGFR2* was a sensitive and reproducible method. We also examined the copy numbers of *FGFR1*, *FGFR3* and *FGFR4*, but no obvious gene amplification was observed in all of the eight cell lines (Figure 1D). Next, *FGFR2* amplification was evaluated using the copy number assay in 267 FFPE samples of primary gastric cancer specimens. *FGFR2* amplification of more than 5 copies was observed in 11 cases (92.0, 63.0, 41.4, 19.9, 18.4, 13.7, 8.3, 6.2, 6.2, 5.7 and 5.6 copies), with a frequency of 4.1% (Figure 2A). The mean copy number in the non-amplified cases was 2.4±0.6 copies. Meanwhile, no obvious gene amplification of *FGFR1*, *FGFR3* or *FGFR4* was observed (data not shown).

### FISH analysis for *FGFR2* amplification

We used a FISH analysis to examine *FGFR2* amplification in the same samples to verify the results of the above PCR-based DNA copy number assay. Highly amplified TU-KATOIII cells showed numerous and large clustered signals, whereas non-amplified OCUM1 cells contained two normally paired signals (Figure 2B). A FISH analysis was performed on seven cases among 11 *FGFR2*-amplified cases and two non-amplified cases. The FISH analysis revealed that *FGFR2* was highly amplified in six of the seven *FGFR2*-amplified clinical samples (four showed multiple scattered signals and two showed large clustered signals), while the remaining sample exhibited a relatively low grade of amplification (FGFR2/CEN10p=2.2, Figure 2B). The *FGFR2* signals in the G3 and G10 samples, which were determined not to be amplified based on the results of the DNA copy number assay, were not increased. These results clearly demonstrated the presence of *FGFR2*-amplified gastric cancers among clinical samples.

### Clinicopathological features of *FGFR2*-amplified gastric cancer

We evaluated the clinicopathological features including age, sex, histology and pathological stage according to the *FGFR2* amplification status. Patients age with *FGFR2* amplification were significantly higher than the others, but sex and pathological stage were not associated with *FGFR2* amplification in this study (Table 1). Among the patients with *FGFR2* amplification, the histologies of two cases were intestinal-type gastric cancer and one was unclassified type, while the others were diffuse-type (Table 2). The tumours were located in either the upper or lower stomach. These results are summarised in Table 2. Finally, we examined the prognostic impact of *FGFR2* amplification on OS after surgery. *FGFR2* amplification tended to be associated with a poorer outcome, compared with non-amplified cases, but no significant difference was observed in the current study (log-rank test, *P*=0.075; Figure 2C).

## Discussion

To date, several studies have reported on the protein expression of FGFR2 and clinicopathological analyses using immunohistochemistry, with 20 of 49 (41%) and 42 of 134 (31%) gastric cancers expressing FGFR2 protein when evaluated using positive or negative staining (Hattori *et al*, 1996; Toyokawa *et al*, 2009). Regarding genomic alteration, the frequency of *FGFR2* amplification has been reported to be 3 out of 19 (16%, among diffuse-type gastric cancers) detected using comparative genomic hybridisation (CGH), 3 out of 57 (5%) detected using Southern blot analysis, and 2 out of 30 (7%) detected using CGH (Tsujimoto *et al*, 1997; Peng *et al*, 2003; Kim *et al*, 2010). These results suggest that the frequency of *FGFR2* amplification is around 5%, which is lower than the positive staining results obtained using immunohistochemistry. However, the frequency of amplification has not been determined in a large cohort. Our results indicated that the frequency of *FGFR2* amplification was 4.1% (11 out of 267), consistent with these previous reports on genomic alterations. To select a sub-population of gastric cancers sensitive to FGFR inhibitors in the future, gene amplification may be a more suitable biomarker than positive staining using immunohistochemistry based on the results of preclinical studies (Figure 1, Takeda *et al*, 2007).

In six cases, the copy number of *FGFR2* was larger than 10 copies and numerous signals were observed by the FISH analysis (Figure 2B), indicating that these gastric cancer cells harboured high levels of amplification, similar to the results obtained using gastric cancer cell lines. Preclinical studies suggest that these cases may be likely to respond to FGFR inhibitors. In the remaining case, *FGFR2* amplification was relatively low (4∼8 copies, G44). Such cases with low levels of *FGFR2* amplification may require further investigation regarding their sensitivity to FGFR inhibitors in the future. Meanwhile, we used a copy number assay to detect gene amplification in FFPE samples. Although DNA extracted from FFPE samples was considered to be of low quality with a DNA degradation in general, a copy number assay was capable of detecting and screening amplification in the FFPE samples, which had been stored for as long as 10 years. The results were consistent with the results of FISH studies in several cell lines, with seven positive cases and two negative cases. Our findings suggest that a copy number assay is a powerful tool for detecting and screening gene amplification using FFPE samples.

Recently, trastuzumab in combination with chemotherapy has been regarded as a new standard option for patients with HER2-positive advanced gastric or gastro-oesophageal junction cancer (Bang *et al*, 2010). Therefore, the evaluation of both the HER2 and FGFR2 status before anti-cancer treatment may be needed in gastric cancer patients in the near future. Many small molecules of VEGFR2 tyrosine kinase inhibitors, categorised as anti-angiogenic agents, are now under clinical evaluation, and some of them, including sorafenib for hepatocellular carcinoma and sunitinib for renal cell carcinoma, are being clinically used as standard treatment options (Ellis and Hicklin, 2008). These compounds are also known to have a potential kinase inhibitory effect on FGFRs (Takeda *et al*, 2007; Turner *et al*, 2010), indicating that the development of these multi-kinase inhibitors may be a promising approach to the treatment of *FGFR2*-amplified gastric cancer. In addition to small molecular FGFR tyrosine kinase inhibitors, anti-FGFR antibodies, such as IMC-A1, PRO-001a and R3Mab, also offer promise as molecular-based drugs (Turner and Grose, 2010). We plan to conduct a prospective study in a cohort of Japanese patients with *FGFR2*-amplified gastric cancers.

In conclusion, we found that *FGFR2* amplification was observed in gastric cancer at a frequency of about 4.1%, and a copy number assay was a powerful tool for screening for *FGFR2* amplifications using FFPE samples. Our results warrant strong consideration of the development of FGFR inhibitors for the treatment of gastric cancers with *FGFR2* amplification.

## Figures and Tables

**Table 1:** Frequency of *FGFR2* amplification in gastric cancers and its association with clinical and pathologic factors

	**FGFR2 (+)**		**FGFR2 (−)**		
	***n*=11**	**%**	***n*=256**	**%**	***P-*value**
*Age*					
Range	55–91		31–88		0.047
Median	67		63		
					
*Gender*					
Male	11	100	173	68	0.052
Female	0	0	83	32	
					
*Pstage*					
I	0	0	25	10	0.16^a^
II	0	0	32	13	
III	3	27	73	29	
IV	8	73	125	49	
Unknown	0	0	1	0	
					
*Histology*					
Tub1	0	0	41	16	0.55^b^
Tub2	2	18	51	20	
Pap	1	9	5	2	
Muc	2	18	8	3	
Sig	1	9	15	6	
Por1	0	0	28	11	
Por2	5	45	108	42	

Abbreviations: Amp=gene amplification; FGFR=fibroblast growth factor receptor; Muc=mucinous adenocarcinoma; Pap=papillary adenocarcinoma; Por=poorly differentiated adenocarcinoma; pStage=pathological stage; Sig=signet ring-cell carcinoma; Tub=tubular adenocarcinoma.

a Comparison between pStage I+II and III+IV.

b Comparison between intestinal (Tub1, Tub2 and Pap) and others. *P*-values were calculated using the *t*-test for age and the *χ*^2^ test for the other variables.

**Table 2:** Summary of *FGFR2*-amplified gastric cancers

**No.**	**Age**	**Sex**	**Location**	**Size of lesion (cm)**	**Macroscopic type** a	**Lauren's classification**	**Histology**	**pStage**	**OS (days)**	**FGFR2 (CN)**	**FISH (type, copies)**
G7	55	M	Lower	8.5 × 8	3	Diffuse	Muc>Por2, Sig	IV	612	41.4	LC, +++
G38	70	M	Upper	8.5 × 8	1 + IIc	Intestinal	Pap>Tub1, Tub2, Por2	IIIa	591	92.0	MS, +++
G44	70	M	Lower	9.5 × 8	3	Diffuse	Por2>Pap, Tub1, Muc	IIIa	938	5.6	Low, 2.2^b^
G46	75	M	Middle	10 × 6	4	Intestinal	Tub2>Por2	IV	2380	13.7	MS, +++
G92	75	M	Middle	6.5 × 5.5	3	Diffuse	Por2>Tub2	IV	280	19.9	MS, +++
G154	59	M	Middle	14 × 12	4	Diffuse	Por2	IV	132	5.7	MS, +++
G163	64	M	Lower	15 × 10	3	Diffuse	Muc>Sig>Tub2	IV	540	6.2	LC, +++
G203	64	M	Lower	10.5 × 6.5	4	Diffuse	Sig>Por2>Muc	IV	283	8.3	ND
G271	91	M	Upper	7 × 6.5	2	Intestinal	Tub2>Por1	IV	383	63.0	ND
G299	65	M	Middle	20 × 20	4	Diffuse	Por2>Sig	IV	256	6.2	ND
G329	67	M	Middle	6.5 × 6	3	Diffuse	Por2>Sig	IIIa	3642+	18.4	ND

Abbreviations: CN=copy number of *FGFR2* determined using a copy number assay; Diffuse=diffuse-type gastric cancer; FISH=fluorescence *in situ* hybridisation; FGFR2=fibroblast growth factor receptor 2; Intestinal=intestinal-type gastric cancer; Location=tumor location in stomach; LC=large clustered signals; Low=low copy number gain; M=male; MS=multiple scattered signals; ND, not determined; No.=sample numbers; OS=overall survival; pStage=pathological stage; +++=numerous *FGFR2* signals; +=patients. alive.

a Macroscopic type, classification is based on the definitions of the Japanese Research Society for Gastric Cancer.

b Ratio of FGFR2/CEN10p.
